# Goldenhar syndrome: the importance of an ophthalmological approach

**DOI:** 10.22336/rjo.2020.68

**Published:** 2020

**Authors:** Pio Guilherme Malta, da Silva Rezende Aline Vilani, Cordeiro Frederico de Miranda, Ibrahim Larissa Fouad, Curi Cláudio Castro, Moura Érica Borgatti

**Affiliations:** * Department of Ophthalmology, Instituto de Olhos Ciências Médicas, Belo Horizonte/ MG, Brazil; ** Department of Ophthalmology, Núcleo Malta, Lagoa da Prata/ MG, Brazil; *** Department of Ophthalmology, AME Excelência em Visão, Congonhas/ MG, Brazil; **** Oculoplastics Department of Eye Plastic, Instituto de Olhos Ciências Médicas, Belo Horizonte/ MG, Brazil

**Keywords:** choristoma, craniofacial microsomia, dermoid cyst, Goldenhar syndrome

## Abstract

The article describes a case of Goldenhar syndrome that had been diagnosed by an ophthalmologist in a medical consultation by school bullying due to a choristoma. A 15-year-old male patient, who had a nodular lesion with hair in the inferior temporal corneal-limbo-conjunctival of the left eye, was reported. He also had a facial asymmetry, with mild mandibular hypoplasia and malformation of the left external ear, where only an auricular appendage was formed. He denied similar family history and the history of genetic diseases, but revealed that his mother had used ibuprofen during the first 3 months of pregnancy and had gestational diabetes mellitus. Excisional biopsy of the eye lesion was performed and revealed a dermoid cyst. After the exegesis and with adequate multidisciplinary monitoring, the patient reported being very satisfied with the aesthetic result, returning with more confidence to his daily activities. That was a typical case of Goldenhar syndrome that has remained undiagnosed and untreated for more than a decade due to a lack of diagnosis despite its classic presentation. The delay in the approach resulted in social stigma and profound psychosocial damage. The importance of disseminating the correct knowledge of this pathology and of having an early multidisciplinary approach in these patients is emphasized, since the impact on the quality of life is significantly high.

## Introduction

Goldenhar syndrome (GS) is a congenital disease that was first described in 1952 by the French ophthalmologist Maurice Goldenhar [**1**,**2**]. Its reported incidence ranges from 1:3,500 to 1:5,600 with a 3:2 ratio between men and women. The unilateral occurrence is about 85%, the right side being more affected than the left in a 3:2 proportion [**1**,**3**]. It has a multifactorial origin, but the etiopathogenesis of the disease is still n1*0.ot well known and probably involves genetic and environmental factors. It may present a pattern of autosomal dominant, recessive inheritance and sporadic forms. It is known that changes in embryonic development occur in the 1st and 2nd brachial arches, as well as occlusive vascular changes in the placental veins [**1**,**2**,**4**].

Classified as a subtype of craniofacial microsomia, it is also known as oculo-auriculo-vertebral dysplasia [**1**,**2**,**4**-**8**]. It is characterized by the classic triad: mandibular hypoplasia with facial asymmetry, oculo-auricular malformations and vertebral abnormalities [**1**,**2**,**4**]. Among other changes, it is possible to observe choristomas, microphthalmia, strabismus, cataracts, coloboma of iris and retina, coloboma of upper eyelid, auricular appendage, asymmetry of the ears, atresia of the auditory external canal, hemifacial microsomia, facial asymmetry, mandibular and/ or maxillary hypoplasia, dental alterations such as supernumerary teeth, Fallot’s tetralogy, dextrocardia, transposition of the great vessels, atresia in the gastrointestinal tract, scoliosis, microcephaly, hydrocephalus, hypoplasia of the corpus callosum and several other alterations described [**1**,**2**,**6**]. Despite the variety of signs and symptoms, the diagnosis is based on clinical examination and complementary tests, such as radiological and blood tests [**4**,**6**].

This article describes a case of Goldenhar syndrome in a 15-year-old male patient without a family history of genetic alterations who, despite presenting characteristics of the syndrome’s ectoscopy, had not yet been diagnosed. Such diagnosis occurred from an ophthalmological care motivated by school bullying due to a choristoma. Given the rarity of this disease, the objective was to disseminate knowledge about this topic in order to facilitate its recognition and encourage early diagnosis and conduct, which can reduce possible negative impacts on the patient’s quality of life.

## Case report

The case of a 15-year-old male patient was reported in the eye plastic department, referenced by the psychologist due to frequent bullying at school motivated by left eye lesion, which made him depressed, introverted and caused successive school failures. The patient described that the lesion was congenital, with slow and progressive growth throughout life. He denied other ophthalmological complaints, ocular traumas, systemic comorbidities and the usage of usual systemic or topical medications. He also denied similar family history and the history of genetic diseases. He had an older brother without any syndromic condition. His gestational history revealed a full-term vaginal delivery, at 38 weeks and 6 days, with no complications. Parents did not have consanguineous marriage, but when asked, revealed that his mother used ibuprofen during the first 3 months of pregnancy and presented gestational diabetes mellitus.

**Fig. 1:**
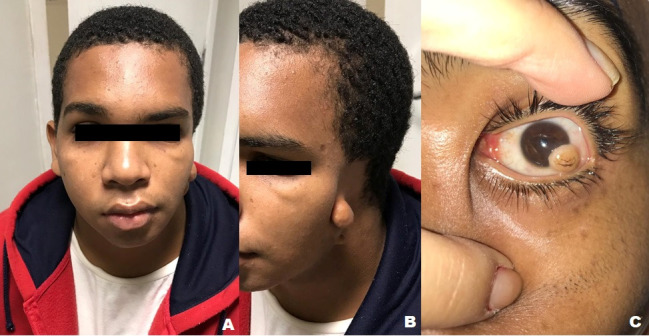
Ectoscopy. A) Facial asymmetry due to mandibular hypoplasia and left ear alteration. B) Absence of auditory pavilion and presence of auricular appendage. C) There is a corneal-limbo-conjunctival dermoid cyst in the left eye, where hair follicles can be noticed

Upon ectoscopy, facial asymmetry was seen, with mild mandibular hypoplasia and malformation of the left external ear, where only an auricular appendage was formed. Eye examination revealed a corrected visual acuity of 1.0 in the right eye (RE) and 0.05 in the left eye (LE). Biomicroscopy of the RE presented without alterations and LE with a nodular, elevated, inferior temporal corneal-limbo-conjunctival lesion, deeply infiltrating the stroma, non-pigmented, with hair follicle in its surface, measuring approximately 5.7 x 5.7 mm (**Fig. 1**). Fundoscopy was normal bilaterally and intraocular pressure within normal limits.

**Fig. 2:**
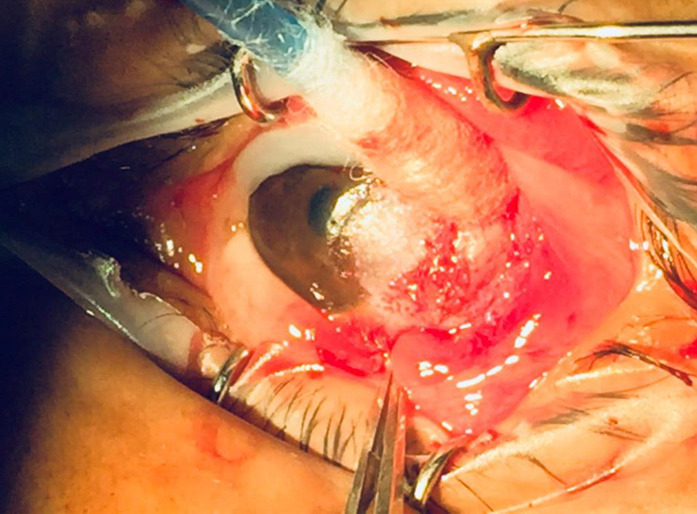
Excisional biopsy. The strong adhesion of the lesion to the deep tissues of the cornea is observed

Due to the characteristics of left eye’s lesion, the diagnostic hypothesis of choristoma was suggested and excisional biopsy was performed, with repair using a conjunctival advancement flap of 10-0 silk suture. Complete excision of the lesion was not possible due to its adherence to the deep corneal stroma (**Fig. 2**). The material was sent for anatomopathological study.

**Fig. 3:**
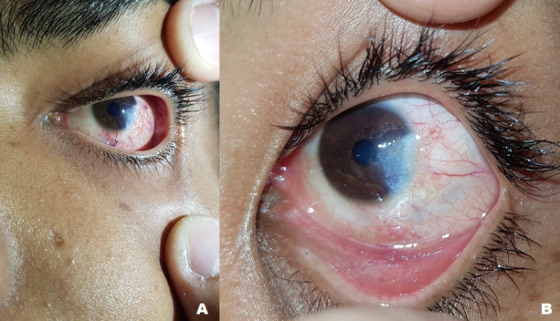
Postoperative appearence. A) 7th day postoperative - 10-0 silk sutures can be observed. B) 12th day postoperative - corneal leukoma at the lesion excision site

On the seventh postoperative day, the left eye had mild ocular hyperemia, partial de-epithelialization, leukoma in the inferior temporal corneal area with the remains of the infiltrative lesion and intact sutures. On the 12th postoperative day, he showed improvement in the de-epithelialized area, staining only punctiform with fluorescein and the sutures were removed (**Fig. 3**). After 3 months of surgery, a calm eye with a small inferior temporal nubecula, local conjunctival covering and a fluoride-negative cornea, was observed. Final best corrected visual acuity remained the same as preoperative, 1.0 and 0.05 in the right and left eyes, respectively, which confirmed amblyopia on the left eye.

The result of the anatomopathological study revealed: “fragments similar to the skin, presenting epidermis with orthokeratosis and good pigmentation in the basal layer keratinocytes. Moreover, the presence of multiple pilosebaceous follicles, being an atrophic follicle with light ectasia, was observed. Presence of a central follicle with discrete hyperplasia of the sebaceous glands showed a mature and well-differentiated aspect. The deep dermis showed groups of merocrine and apocrine sweat glands”.

Dental, otorhinolaryngological and orthopedic evaluations were requested, which showed abnormalities such as: mandibular dysgenesis, absence of the duct and auditory pavilion on left ear and vertebral hypoplasia.

Due to the presence of a typical choristoma, as well as the presence of all the other clinical signs, Goldenhar syndrome clinical diagnosis was made. Genetic tests were requested for confirmation, but the present location did not have this service and the patient was not willing to pay for the exam on his own.

The patient was very satisfied with his final aesthetic result and was then referred for evaluation of plastic surgery in order to correct the other anatomical alterations. He returned to his daily activities and remained under psychosocial follow-up. The psychological representative sent a new report of his psychological evaluation describing an important improvement in his depression, introversion and lack of self-confidence.

## Discussion

GS is well known for its classic triad: mandibular hypoplasia with facial asymmetry, oculo-auricular malformations and vertebral abnormalities. The phenotype of each affected individual can vary greatly in severity, depending on the activation and degree of penetration of the defective gene. Among ocular manifestations, epibulbar choristoma is present in one third of the patients with SG, with the unilateral occurrence about 50% [**3**,**5**,**6**]. Other common manifestations such as microphthalmia, lipodermoids and coloboma, strabismus, cataracts, and epicanthic folds are also described together with anophthalmia, anomalies of the lacrimal drainage system, anomalies of the retina, optic nerve and glaucoma [**2**,**3**,**5**]. In addition to ocular changes, the association of Goldenhar syndrome with facial microsomia, maxillary and mandibular hypoplasia, malar flattening and the presence of ear tags occurrence, are common in about 80-90% of the cases [**2**,**3**]. 

Other alterations are also described, such as low auricular implantation, laryngomalacia, cleft lip or palate, anomalies of vertebrae such as hemivertebrae and scoliosis, congenital heart disease, such as ventricular septum and tetralogy of Fallot, renal and gastrointestinal changes, among others [**1**,**3**,**4**,**7**]. 

Our patient presented typical manifestations of the syndrome, including the classic triad, despite the less common involvement on the left side, as a mandibular hypoplasia with facial asymmetry, vertebral changes and ocular-auricular changes, such as choristoma and absence of formation of the outer and middle ear. However, despite the clinical diagnosis, the genetic study was not performed due to the impossibility of access by the patient.

The etiopathogenesis of the disease is still not well known and the hypothesis of a multifactorial etiology involving nutritional and environmental factors together with genetic dysregulation is accepted [**2**,**3**,**7**]. However, in most cases, the syndrome is sporadic [**2**,**3**,**5**]. 

A defect in the embryonic period with imbalance in cells during blastogenesis affects the derivatives of the first and second brachial arches explaining the manifestations of the syndrome [**8**]. Chromosomal aberrations such as *3del (5p), del (6q), del (8q) (161), del (18q), del (22q)*, recombinant chromosome 18, ring chromosome 21 and dup (*22q*), maternal and fetal hypoxia, hypertension, maternal diabetes, influenza or rubella viral infection, malnutrition, smoking, radio-diagnostic procedures between the 4th and the 6th week of pregnancy, exposure to drugs such as cocaine, tamoxifen, ibuprofen, retinoic acid and consanguineous marriage are possible predisposing factors [**1**,**3**,**4**,**6**,**7**]. 

Reassessing the history of our patient, some risk factors for the occurrence of the syndrome were presented, such as the history of gestational diabetes mellitus and the use of ibuprofen during the first trimester of pregnancy. However, there was not any report of a history of genetic syndromes in the patient’s family. Therefore, it was believed that it was possibly a sporadic manifestation, our patient being the first case to occur in his family.

## Conclusion

This article described a case of Goldenhar syndrome that remained more than a decade without investigation, diagnosis, treatment and adequate multidisciplinary follow-up, even causing significant permanent damage to the visual acuity of the left eye. Because its clinical characteristics were exuberant and easily visible at ectoscopy, GS was not a major diagnostic challenge.

The patient had serious psychosocial consequences due to the stigma caused by school bullying that resulted in social and educational damage. Early diagnosis could have helped prevent this social stigma in addition to early intervention trying to avoid amblyopia, depression, low self-esteem, school failures and social damage.

The importance of knowing the syndrome in its broad spectrum of changes was therefore emphasized. The extreme relevance of the multidisciplinary approach in these patients was also emphasized, since the impact on quality of life was significantly high.

**Conflict of Interest**

None. All authors have no conflict of interest in this work. All authors contributed equally to this work.

**Informed Consent**

The authors certify that they have obtained all appropriate patient consent forms. The patient gave his consent for his images and other clinical information to be reported in the journal, in the signed form. The patient understood that his name and initials will not be published and due efforts will be made to conceal his identity, but anonymity cannot be guaranteed.

**Authorization for the use of human subjects**

The research related to human use complies with all the relevant national regulations, institutional policies, is in accordance with the tenets of the Helsinki Declaration, and has been approved by the Ethics Committee of the Eye Plastic Department of Instituto de Olhos Ciências Médicas.

**Acknowledgements**

 None.

**Sources of Funding**

None.

**Disclosures**

None.
